# Tuning the Charge of Sliding Water Drops

**DOI:** 10.1021/acs.langmuir.2c00941

**Published:** 2022-05-02

**Authors:** William S. Y. Wong, Pravash Bista, Xiaomei Li, Lothar Veith, Azadeh Sharifi-Aghili, Stefan A. L. Weber, Hans-Jürgen Butt

**Affiliations:** Max Planck Institute for Polymer Research, Ackermannweg 10, D-55128 Mainz, Germany

## Abstract

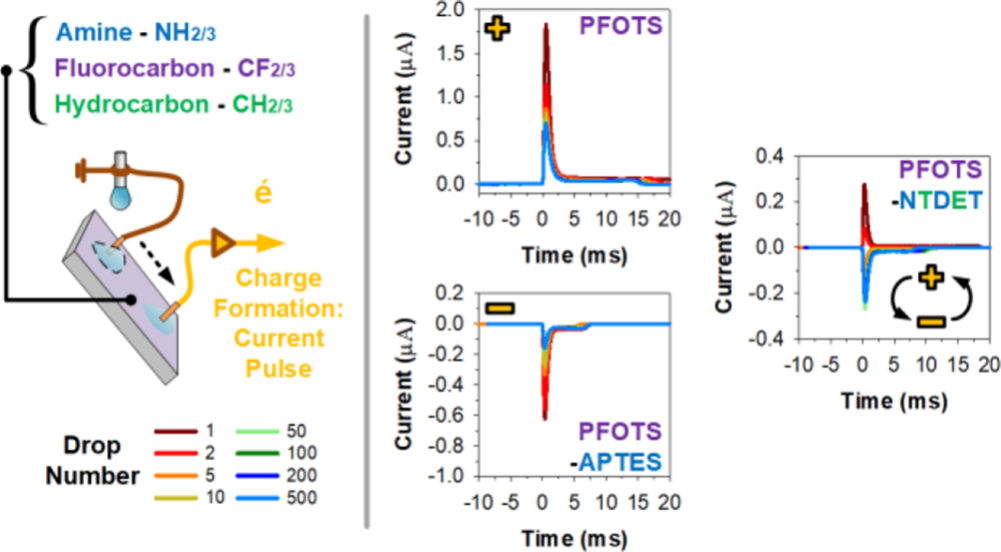

When a water drop slides over a hydrophobic surface, it usually acquires a positive charge and deposits the negative countercharge on the surface. Although the electrification of solid surfaces induced after contact with a liquid is intensively studied, the actual mechanisms of charge separation, so-termed slide electrification, are still unclear. Here, slide electrification is studied by measuring the charge of a series of water drops sliding down inclined glass plates. The glass was coated with hydrophobic (hydrocarbon/fluorocarbon) and amine-terminated silanes. On hydrophobic surfaces, drops charge positively while the surfaces charge negatively. Hydrophobic surfaces coated with a mono-amine (3-aminopropyltriethyoxysilane) lead to negatively charged drops and positively charged surfaces. When coated with a multiamine (*N*-(3-trimethoxysilylpropyl)diethylenetriamine), a gradual transition from positively to negatively charged drops is observed. We attribute this tunable drop charging to surface-directed ion transfer. Some of the protons accepted by the amine-functionalized surfaces (−NH_2_ with H^+^ acceptor) remain on the surface even after drop departure. These findings demonstrate the facile tunability of surface-controlled slide electrification.

## Introduction

Charge formation on solid–gas interfaces after liquid contact (i.e., charge separation^1,2^) is a universal but incompletely understood phenomenon.^3^ Charge separation is commonly attributed to ion transfer to the solid because of the presence of ions, such as H^+^ and OH^–^ in water.^4−6^ During the sliding contact of water drops on surfaces, charging (i.e., slide electrification) is induced by sequential ionization, dissociation, and adsorption of ions onto the solid surface. The charges are then left on the surface after departure of the drop. Thereafter, they are neutralized by the environment,^7^ which may be caused by the flow of electrons, ion migration, and/or desorption of ions.^8,9^ However, despite significant research in charge separation and slide electrification, much dispute still remains.^10,11^ In particular, the origin of charge formation can still be attributed to ion^8,11−16^ and/or electron^10,11,16,17^ transfer.

The aim of this work is to improve the understanding of ion-transfer mechanisms by changing the surface chemistry of hydrophobic surfaces. Based on this knowledge, we intend to control the strength and polarity of slide electrification. To date, almost all slide electrification experiments have produced positively charged drops and negatively charged surfaces.^8,10−12,14,18−20^ The rare exception of producing negatively charged drops was achieved only through the use of low pH (pH < 3) by Sosa et al.,^12^ thus below the surface’s pH of zero charge, that is, the pH at which the net charge of the surface is zero.

To our knowledge, the use of hydrophilic or water-interactive functionalities (i.e., amines) was never achieved under continuous drop slide electrification. Hydrophobic surfaces are often used because drops do not easily slide on hydrophilic surfaces. Sliding drops become unstable, and satellite droplets are formed.^21^ The formation and presence of remnant satellite droplets affects the charging–discharging dynamics and the continuation of drop slide electrification (i.e., the surface eventually appears uncharged as bulk water actively discharges surfaces). To exploit the use of hydrophilic moieties (i.e., amines), a two-step functionalization process is used. In the first step, a hydrophobic (hydro/fluorocarbon) primary layer is functionalized. In the second step, the sequential functionalization of secondary amine layers confers charge modification characteristics. These amine-integrated hydrophobic surfaces preserve low contact angle hysteresis and inhibit the formation of satellite droplets during slide electrification. The integrated amines experience a hydrolytic reaction during wetting, consuming H^+^ ions, R-NH_2_ + H^+^ → R-NH_3_^+^, thus producing negatively charged water drops.

## Experimental Section

### Chemical Vapor Deposition Synthesis of Surfaces

#### Cleaning and Activation of Amorphous Glass Substrates

Amorphous glass substrates (1 mm thick) (Thermoscientific) were washed in streams of ethanol and acetone before blow-drying by dry nitrogen. Care was taken to avoid the use of visually evident defective glass (stains, spots, cracks, etc.). The glass substrates were then O_2_-plasma-treated for 10 min at 100% power (Diener Electronic Plasma Surface Technology: Femto BLS, Ebhausen, Germany).

#### Primary Functionalization Layers

Cleaned and activated glass substrates were placed into a desiccator (25 cm diameter, *V* = 9.2 L) at a distance of ca. 11 cm from the center, where 1 mL of the functionalizing agent was deposited, ca. 1 cm lower than the glass substrates. Silanes were deposited into the desiccator before evacuation. The hydrocarbon-based trichloro(propyl)silane, TCPS, was evacuated to ca. 200 mbar while the fluorocarbon-based trichloro(1*H*,1*H*,2*H*,2*H*-perfluorooctyl)silane, PFOTS, was evacuated to ca. 50 mbar. A reaction time of 30 min was allowed for primary layers. A separate desiccator was used for each chemical (TCPS and PFOTS) to avoid cross-contamination.

#### Secondary Functionalization Layers

If the primary layers are to be integrated with a secondary layer (see below), only 5 min (TCPS) and 10 (PFOTS) min of reaction time was allowed, respectively. Then, secondary layers of (3-aminopropyl)triethoxysilane, APTES or *N*-(3-trimethoxysilylpropyl)diethylenetriamine, NTDET, were deposited as 1 mL of liquid into their own respective desiccator, again at ca. 11 cm from the center. The pressure was then evacuated to ca. 50 mbar and kept for 6 h.

#### Completion of Functionalization and Equilibration Time

After functionalization, the glass substrates were kept at 50 mbar without any silane present to vent unreacted silanes. Afterward, the coated glass substrates were left to equilibrate with the ambient air environment (*T* = 20 °C, humidity = 30–70%) for 5 days before testing. Because of the tri-functionality of silanes used,^29−31^ sequential surface hydrolysis and condensation reactions will result in a cross-linked network with both material configurations layered.

### Charge Analysis

The charge measurement setup is kept in an electrically grounded Faraday cage, as described before.^7^ Peripheral electrical devices, that is, the pump and the current amplifier were kept outside of the Faraday cage. Test surfaces were mounted on a tilted stage at an angle of 50°. A flat-tipped syringe needle, 2 mm inner diameter, was mounted 5 mm above the top part of the sample. A peristaltic pump (Gilson Minipuls 3, Wisconson, USA) was used to produce *V* = 45 μL deionized water drops (Sartorius Arium Pro VF, 18.2 MΩ cm resistivity, Germany) at an interval of between 2 and 16 s. The drops fell approximately 0.5 cm, at minimum natural detachment height, before landing on the coated glass surfaces. As drops slide down, they contact a series of two electrodes. The first electrode (silver wire, upon landing) grounds the drop. The second electrode (gold-tipped electrode, at 4 cm) measures the drop current via a low noise current amplifier (response time: 5 ms, FEMTO DLPCA-200, Berlin, Germany). Upon leaving the second electrode, drops roll over the extended end of the coated glass surface, and into a collection dish. Before every new experiment, an ionizing air stream (Simco-Ion, Pennsylvania, USA) was directed over the surface for 1 min in order to neutralize the surface. Data were collected and recorded using a National Instruments data acquisition card (NI USB-6366 X-Series) and LabVIEW software. Drops (typically 500 drops) run successively over the coated glass surface at defined time intervals (typically 2 s). For every drop, a current spike was recorded using the second electrode. Current signals were integrated for every drop to quantify the drop charge. Repeats are typically performed over 3–5 days of cross-batch samples and multiple repeats on each day (up to 3 readings), culminating in a total of 10 data points for each surface variant analyzed.

### Wetting Analysis

Roll-off angle, and advancing and receding contact angles were measured using 45 μL water drops using an OCA 35 contact angle goniometer (Dataphysics, Germany, zoom factor 1.0). Drops were tilted (1°/s) until they rolled off. The contact angle hysteresis and roll-off angles are reported respectively for the surface variants over three measurements.

### Drop Mobility Analysis

To measure the dynamics of sliding drops, 33 μL water drops were deposited at intervals of 2 s on tilted surfaces. The syringe needle was grounded and kept at 5 mm above the surfaces. A peristaltic pump (Minipuls 3, Gilson) delivers the drops. Drops were imaged at a frame rate of 1000 FPS (FASTCAM MINI UX100, Photron with a Titan TL telecentric lens, 0.268×, 1″ C-Mount, Edmund Optics), from the front and the side over a slide length of 4 cm. Analysis of the video images allows extraction of drop velocity and advancing/receding contact angles. All parameters vary with time and position. To extract advancing/receding contact angles, the open drop shape analysis package from MATLAB (DSAfM) is adopted. Dynamic contact angles were determined by applying a polynomial fit to every contour image.

## Results and Discussion

### Surface Functionalization and Characterization

**Figure 1:**
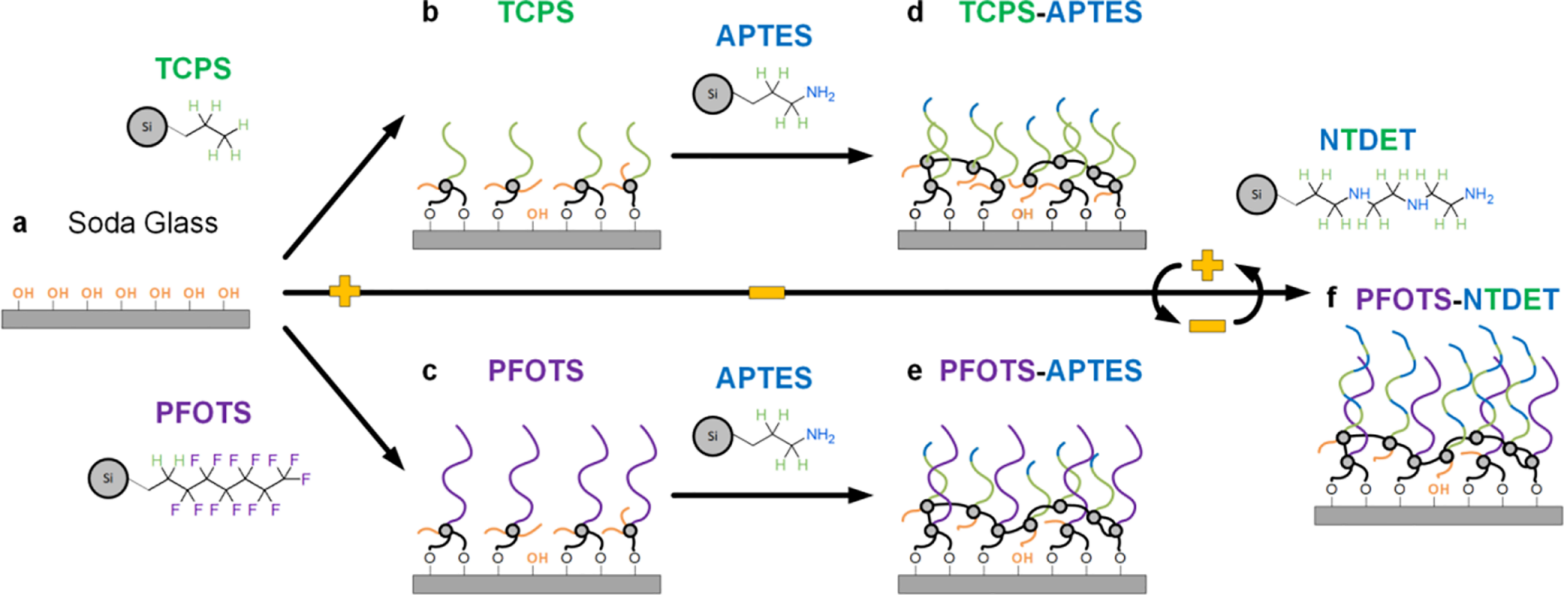
Concept: bi-layered surface functionalization. To inhibit wetting, (a) plain soda lime glass was functionalized with a primary layer of (b) hydrocarbon (trichloro(propyl)silane, TCPS) or (c) fluorocarbon (trichloro(1*H*,1*H*,2*H*,2*H*-perfluorooctyl)silane, PFOTS). Thereafter, secondary layers of (d,e) (3-aminopropyl)triethoxysilane, APTES, or (f) *N*-(3-trimethoxysilylpropyl)diethylenetriamine, NTDET, were added. The relevant functional molecular groups are hydrocarbon: green, fluorocarbon: purple, amine: blue, and unreacted hydroxyls: orange, respectively. The (+) and (−) symbols indicate the nature of polarity during slide electrification.

Plain soda lime glass was functionalized (see the Experimental Section) either by a primary layer of trichloro(propyl)silane (TCPS) or trichloro(1*H*,1*H*,2*H*,2*H*-perfluorooctyl)silane (PFOTS) (Figure 1). Thereafter, a secondary layer of a mono-amine: (3-aminopropyl)triethoxysilane (APTES) or a multi-amine: *N*-(3-trimethoxysilylpropyl)diethylenetriamine (NTDET) was added. In contrast to the simpler mono-amine, NTDET has an alternating molecular configuration as follows: a −C_3_H_6_–, a secondary amine −NH–, followed by −C_2_H_4_–, a second secondary amine −NH–, followed by −C_2_H_4_–, and a primary terminal amine −NH_2_.

The contact angles (advancing and receding) observed on TCPS-functionalized (hydrocarbon) surfaces are slightly lower than those on the PFOTS-functionalized (fluorocarbon) surfaces (Table 1). When integrated with the secondary layer of APTES or NTDET, the contact angles did not decrease but even show a slight increase, despite the fact that the pure APTES or NTDET layers are not hydrophobic. Atomic force microscopy (AFM) measurements (Figure S1 and Table 1, last column) show that the hydrocarbon (TCPS)- and fluorocarbon (PFOTS)-functionalized primary layers are composed of a homogeneous layer and bumps that are up to 10–20 and 30–45 nm high (Figure S1), respectively. Secondary amine (APTES/NTDET) layers are much smoother (<5 nm textures). During repeated sliding drop contact (i.e., 500 drops), these macroscopic nanobumps remain. A complementary time-of-flight secondary ion mass spectrometry (TOF-SIMS) analysis (Figure S2 and Supplementary Discussion) shows that the amine-integrated surfaces have mixed chemical compositions (both amine- and hydro/fluorocarbon) despite minimal wettability variations from purely hydrophobic surfaces.

**Table 1:** Dynamic Wetting Analysis on Both Primary (Hydro/Fluorocarbon)- and Secondary (Amine)-Functionalized Surfaces^a^

Surface Variant	Advancing CA	Receding CA	Roll-Off CAH	Roll-Off Angle	RMS Roughness (nm)
TCPS	92 ± 1	73 ± 1	19 ± 1	17 ± 1	3.0 ± 0.6
PFOTS	107 ± 1	89 ± 2	17 ± 2	21 ± 2	6.9 ± 1.0
APTES	67 ± 3	33 ± 1	34 ± 4	25 ± 3	0.9 ± 0.3
NTDET	67 ± 5	35 ± 2	33 ± 6	31 ± 7	0.5 ± 0.1
TCPS-APTES	94 ± 1	77 ± 3	9 ± 1	16 ± 1	5.7 ± 0.6
PFOTS-APTES	111 ± 1	102 ± 2	18 ± 1	18 ± 1	7.9 ± 1.7
PFOTS-NTDET	113 ± 4	91 ± 4	22 ± 4	30 ± 6	5.7 ± 1.6

a Wetting analysis (roll-off angle and contact angle hysteresis) was performed using 45 μL water drops that were tilted at 1°/s until rolling off.

### Slide Electrification

**Figure 2:**
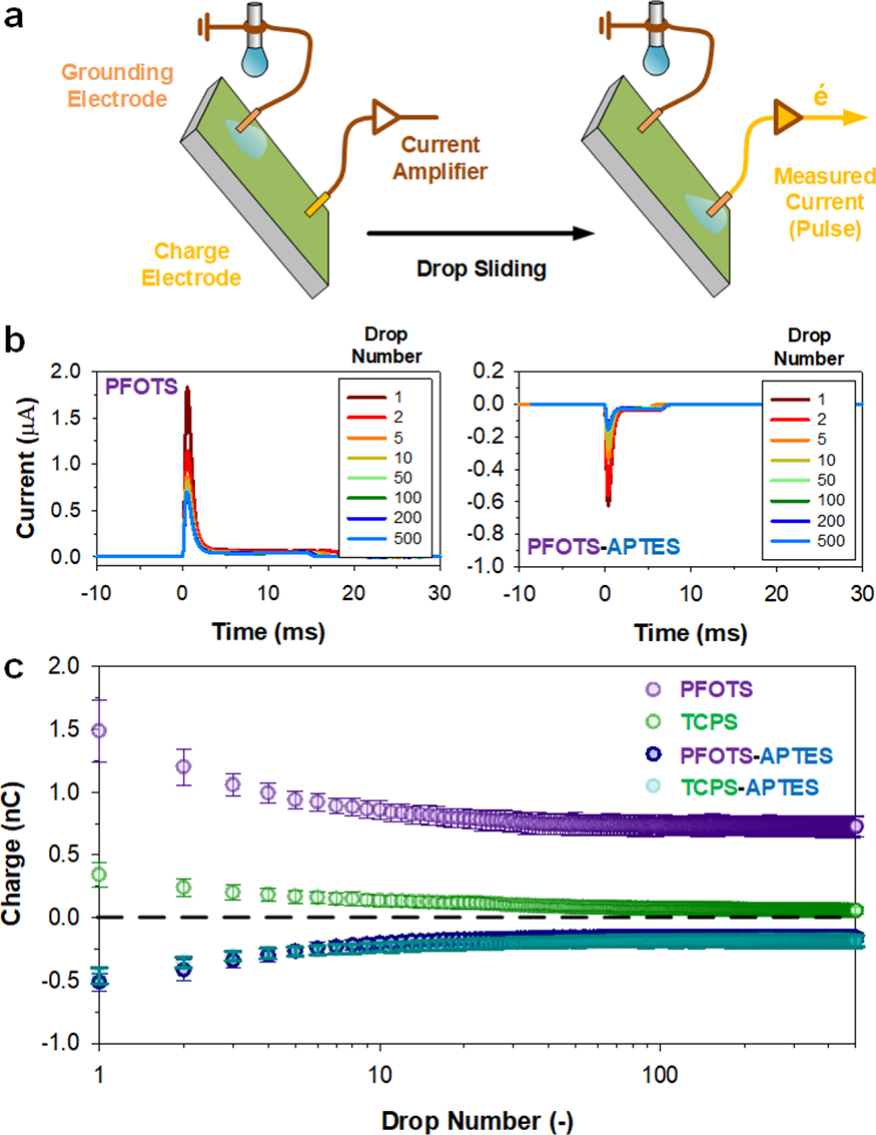
Tunable slide electrification. Slide electrification is achieved by sequentially dropping-and-sliding liquid water drops on tilted, functionalized glass substrates. (a) The drop current is measured using an electrode, which is then amplified for analysis. (b) Depending on the surface, the charges collected can be positive (hydro/fluorocarbon, as shown: PFOTS-functionalized) or negative (amines, as shown: PFOTS-APTES-functionalized). (c) The drop charge saturation for all variants is achieved within 500 drops (10 repeat runs, presented as average ± standard deviation). Both pure hydrophobic variants (PFOTS, purple, and TCPS, green) show positive initial and saturation charges. Secondary functionalization of the mono-amine (APTES) with both hydrophobic variants (PFOTS-APTES, blue, and TCPS-APTES, cyan) results in negative drop charging, showing both negative initial and saturation charges.

Slide electrification experiments^7^ were performed (Figure 2a) using deionized water (≈pH 5). Drops (45 μL) were deposited at a height of 0.5 cm on surfaces tilted at an angle of 50°. Drops were discharged with the first electrode (ground electrode) directly after landing. Drops then slide for 4 cm. The sliding drop collects charges from the contacting surface as it moves. This charge is accumulated until the drop reaches a second electrode (charge electrode) where it discharges the drop as a pulse (width of 2.5 ms, Figure 2b). The pulse travels through a current amplifier which collects and amplifies the charges for measurements. Drop charge is obtained by integrating the current signal (Figure 2b). The fixed drop size (*V* = 45 μL), drop interval (Δ*t* = 2 s), and slide length (*x* = 4 cm) ensure that measurements across surfaces are consistent. Repeats were performed over 10 separate runs (3–5 sampling batches) and averaged (Figure 2). Further details are included in the Experimental Section.

### Positive Charging

The first drop sliding down the surface carries the highest charge. For subsequent drops, the charge decreases and reaches a steady state after ca. ten drops. At the steady state, incoming drops charge the surface as quickly as the surface discharges. On hydrocarbon (TCPS)- or fluorocarbon (PFOTS)-functionalized surfaces, drops charge positively (Figure 2c, green: TCPS, purple: PFOTS).^7,8,10−12,14,18−20^ Hydrocarbon (TCPS) surfaces charge lower, at an initial charge of ca. 0.5 nC, which saturates to ca. 0.05 nC ± 0.03 nC. The fluorocarbon (PFOTS) variant charges higher, at an initial charge of ca. 1.5 nC, which saturates to ca. 0.71 nC ± 0.08 nC. Current curves for PFOTS are also included in Figure 2b, left panel. This positive drop electrification effect is widely attributed to the autolysis of water on hydrophobic surfaces and the adsorption of the hydroxide ion, OH^–^. We assume that a part of these adsorbed OH^–^ ions do not recombine at the receding side of the drop but remain on the surface. A net surplus of protons, H^+^, is thus found in the departing drop.^14,22^ The phenomenon of charging highly positive drops on PFOTS is well-known,^11,12,14^ with more recent studies^18,19^ corroborating the higher charging capacity of fluorocarbon *vs* hydrocarbons or siloxanes.

### Negative Charging

After the secondary-functionalization by a mono-amine (APTES), the hydrophobic surfaces (TCPS and PFOTS) charge contacting drops negatively (Figure 2c, cyan: TCPS-APTES, blue: PFOTS-APTES). Drops charge negatively (from the onset) and at similar magnitudes for both hydro- and fluoro-carbon-based surfaces (TCPS-APTES and PFOTS-APTES). The initial charge was ca. −0.6 nC with a charge saturation of ca. −0.18 nC ± 0.04 nC (TCPS-APTES) and −0.15 ± 0.03 nC (PFOTS-APTES). The magnitudes of the average initial and saturation drop charges were almost identical, regardless of the primary sublayer (TCPS or PFOTS). Current curves are also included for PFOTS-APTES in Figure 2b, right panel. We note that pure APTES surfaces cannot be used under these test conditions as drops fragment during sliding.

The polarity flip induced by the functionalization of the mono-amine (APTES) is attributed to the active hydrolytic protonation during contact with water. This reaction consumes H^+^ ions from the sliding drop, R-NH_2_ + H^+^ → R-NH_3_^+^, thus resulting in negative drop electrification. To our knowledge, this is the first instance where continuous water drops of normal pH are shown to charge negatively during slide electrification.

### Adaptive (Positive-to-Negative) Charging

**Figure 3:**
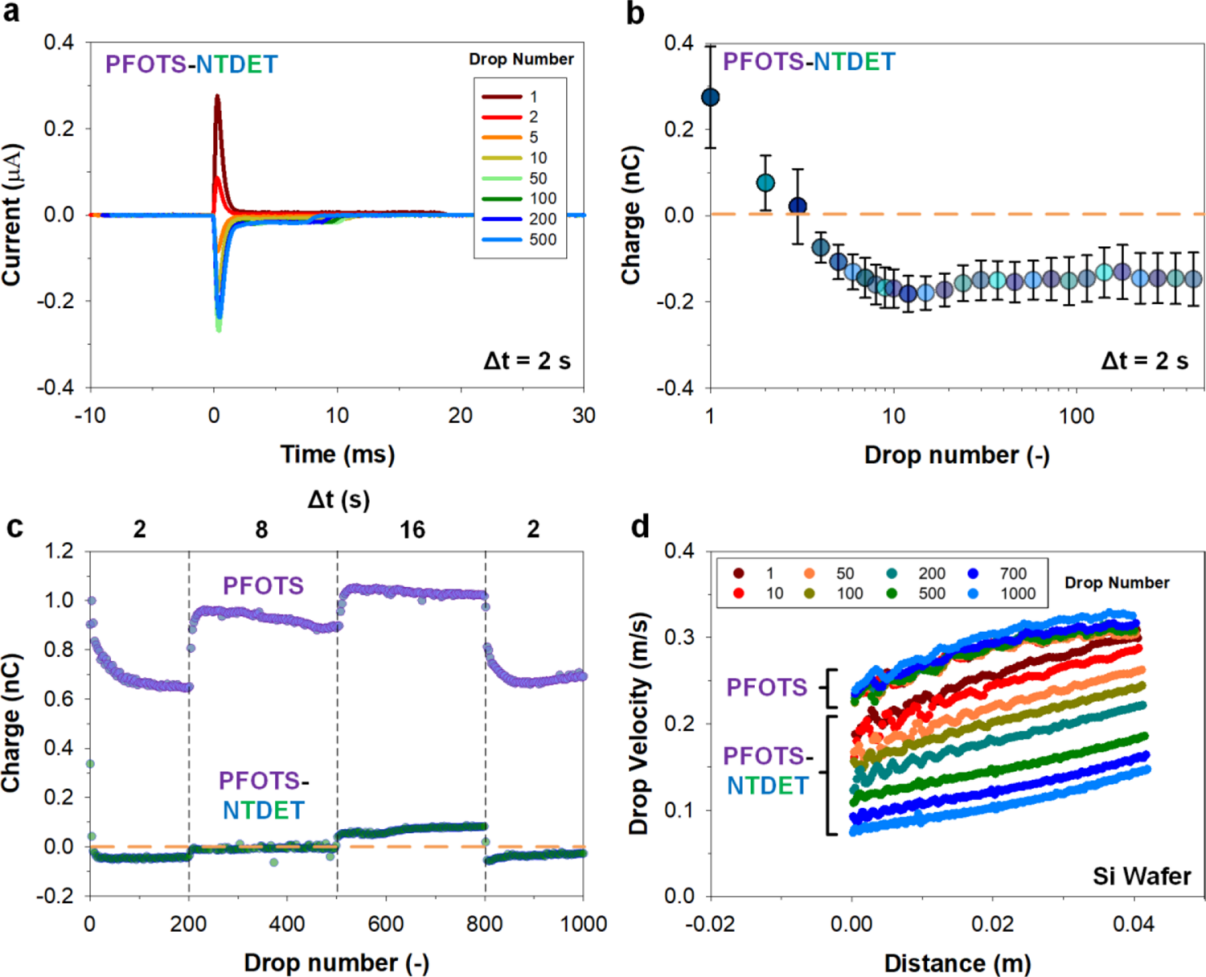
Adaptive charging phenomenon (PFOTS-NTDET). Sequential drop deposition (45 μL, 2 s interval) shows an (a) initial positive drop charge but adapts and saturates at a negative drop charge equilibrium. (b) The positive charge persists for ca. 3 drops before flipping toward a negative charge. The drop charge saturation is achieved, albeit first showing a minor overshoot, within 50 drops (10 repeat runs, presented as average ± standard deviation). (c) The positive-to-negative drop charging adaptation in PFOTS-NTDET is reversible (dark blue), with a longer drop interval (top *x*-axis, in seconds) enabling reversal back to a positive charging behavior. (d) Drop sliding velocity was analyzed using a conductive silicon wafer substrate that removes the influence of charging on drop mobility. Without the influence of electrostatics, and over the course of 1000 sequential drops (33 μL), drop mobility adapts (i.e., changes), indicative strongly of surface chemistry adaptation. (c, d) PFOTS (purple) is included as a control for comparative purposes.

On the NTDET functionalized PFOTS surfaces (PFOTS-NTDET), we observe a rapid decrease in charge magnitude which eventually leads to a flip in the drop charge polarity (to negative values) after 3−4 drops (Figure 3a): the initial drop charge is positive between ca. 0.2 and 0.4 nC, turning negative at ca. −0.15 nC ± 0.03 nC. Saturation of equilibrium charge is complete after ca. 50 drops, after a minor overshoot at ca. 10 drops (Figure 3b).

This polarity flip of PFOTS-NTDET surfaces may be attributed to surface adaptation.^23−25^ Adaptive surfaces can show a reversible alteration of their physical properties under exposure to external stimuli (e.g., wetting). To demonstrate the reversibility of the charge polarity flip, the drop interval (Δ*t*) was increased every 200 drops from the initial 2 s up to 16 s (Figure 3c, PFOTS-NTDET). Under these conditions, the initial positive-to-negative transition (Δ*t* = 2 s) reverses at a drop interval of Δ*t* = 16 s. At the end of the negative-to-positive reversal, the surface could be made to recreate negative drop charging by reshortening drop intervals (Δ*t* = 2 s). Notably, the initial positive surface charge can also recover by letting the surface dry for several minutes. In contrast, control PFOTS surfaces do not exhibit this characteristic reversible flip in charge polarity (Figure 3c, PFOTS).

To confirm wetting-induced adaptation for PFOTS-NTDET surfaces, drop mobility can be analyzed. However, the influence of charging on drop mobility must first be removed. To this end, a silicon substrate was employed (Figures 3d and S3). The high permittivity of silicon substrates reduces electrostatic forces, thus eliminating the influence of charging.^26^ The surface preparation was identical to that on glass. One thousand drops (33 μL) were then deposited (Δ*t* = 2 s) on the functionalized silicon substrates at a tilt angle of 50° (i.e., charge measurement conditions). On PFOTS-NTDET surfaces, we observe large changes in drop mobility. Drops significantly slow down (ca., 2–3 times) over the course of 1000 sliding drop events (Figure 3d). This change in drop-on-surface mobility without the influence of electrostatic forces strongly indicates the presence of surface adaptation.

Such adaptation is not seen in control experiments with PFOTS (Figure 3d, PFOTS) or PFOTS-APTES-coated silicon wafers (Figure S3). Changes in surface chemistry can influence drop mobility by affecting wettability (hydrophilicity), thus surface adhesion, and subsequently the drop profiles and contact line behaviors. Here, the detected drop profiles (i.e., drop length, Figure S4) are notably altered in PFOTS-NTDET vs PFOTS during continuous drop contact. There appears to be minimal changes in contact angles (advancing or receding, Figure S4), but this may be due to the detection limit of the optical method employed. Any changes in receding contact angles at a sub-10 μm scale may be undetectable. Based on these collective observations, surface adaptation is likely, which influences the resultant dynamic drop profile, hence adhesion and mobility.

### Possible Mechanisms in Polarity-Flipping: Adaptation and Charging Kinetics

With the multiamine NTDET, the presence of more amine functionalities should promote further protonation events, secondary R-NH-R + H^+^ → R-NH_2_^+^-R and primary R-NH_2_ + H^+^ → R-NH_3_^+^. However, the observed charging of the multiamine did not lead to a larger magnitude in negatively charged drops during slide electrification. Instead, surfaces experience the as-observed flip in charge polarity. This effect can be tentatively explained using a combination of structural adaptation and/or charging kinetics.

One possible explanation of the flip in polarity is to assume that in its pristine state, the amine-functional groups of NTDET are covered by hydrophobic (C_*x*_H_*y*_ or PFOTS’s C_*x*_F_*z*_) moieties at the solid–air interface. These hydrophobic groups capture negative charges, *q*_1_^–^, that is, hydroxyl (OH^–^) ions, thus leading to temporally positive charges (1–3 drops) during initial wetting. During continuous drop sliding/wetting (>1 drop), the hydrophilic NH_1/2_ groups begin to capture positive charges or protons (H^+^), *q*_2_^+^, in a protonation reaction. From a structural adaptation perspective, hydrophilic NH_1/2_ groups can even reorientate to enrich at the water interface, replacing the originally surface-dominant hydrophobic C_*x*_H_*y*_ or C_*x*_F_*z*_ groups.^27,28^ These structural changes further enhance protonation (increasing *q*_2_^+^).

With the monoamine, PFOTS-APTES, positive drop/negative surface charging is never observed (i.e., net *q*_2_^+^ > net *q*_1_^–^). This is likely due to the shorter hydrocarbon chain in APTES (C_3_N_1_) vs NTDET (C_7_N_3_). In NTDET, the flexibility of the longer chains may allow a certain degree of molecular-level shielding, with hydrocarbons covering amines (at the solid–air interface) in the pristine dry state, thus allowing for an initial onset of positive drop charging (albeit temporally unstable). In APTES, the shorter chains are more rigid and may not permit shielding, with exposed amines immediately inducing negative drop charging. This hypothesis can tentatively explain our experimental observations: the longer hydrocarbon chain of the multiamine NTDET may have provided a secondary (wetting-sensitive) negative charge capture characteristic, *q*_3_^–^, in contrast to that in which APTES does not confer.

As a consequence, the PFOTS-NTDET surface charges via *q*_net_ = *k*_1_*q*_1_^–^ + *k*_2_*q*_2_^+^ + *k*_3_(*t*) · *q*_3_^–^ instead of *q*_net_ = *k*_1_*q*_1_^–^ + *k*_2_*q*_2_^+^ as exhibited by PFOTS-APTES. *k*_1 – 2_ are constants with minimal adaptivity, while *k*_3_ is wetting time-dependent, completely decaying (to a negligible level) within a few seconds of wetting. Drops are charged at −*q*_net_. Therefore, if the PFOTS-NTDET surface is wetted with an increased drop interval (i.e., Δ*t* = 16 s), the reacted amines can deprotonate and/or hydro/fluorocarbon groups can reorientate. *k*_3_(*t*) returns to its finite pristine value. As a result, drop charging returns to positive, that is, polarity reversal. The combination of structural adaptation and/or charging kinetics tentatively provides a possible mechanism behind the as-observed net negative or positive drop charging behavior at low or high drop intervals (Δ*t*), respectively.

## Conclusions

Water drops sliding over hydrophobic layers doped with amine groups acquire a negative drop charge. This was achieved by coating glass surfaces using sequential chemical vapor deposition of fluoro- or hydrocarbon silanes followed by amine-functionalized silanes. This coating method preserved hydrophobicity while conferring the amine-terminated functionality. The amine groups charge positively in water, R-NH_2_ + H^+^ → R-NH_3_^+^. We believe that part of this charge remains on the surface even after the drop has departed. When using a short-chained mono-amine, APTES, drops charge immediately and saturate negatively. With a longer-chained multiamine, NTDET, the polarity of the saturation drop charge depends on the time interval between drops. These surface-directed polarity switching designs depict guidelines toward the future of tunable drop slide electrification.

## Supporting Information Available

(1) Descriptions of supplementary experiments, (2) supplementary discussion on TOF-SIMS analysis of surface chemical compositions, (3) AFM analysis of surfaces, and (4) supplementary drop mobility experiments (PDF)
