# Supplementary material for: Tuning the Charge of Sliding Water Drops
Source: Langmuir. 2022 May 2;38(19):6224–30. doi: 10.1021/acs.langmuir.2c00941 (PMC9118544; doi:10.1021/acs.langmuir.2c00941)
Supplement: Supplementary file 1 — la2c00941_si_001.pdf [file la2c00941_si_001.pdf]

Supplementary Materials for

Tuning the Charge of Sliding Water Drops

William S. Y. Wong, Pravash Bista, Xiaomei Li, Lothar Veith, Azadeh Sharifi-Aghili, Stefan A. L. Weber, and Hans-Jürgen Butt**

Max Planck Institute for Polymer Research,
Ackermannweg 10, D-55128, Mainz, Germany

Keywords: Surface chemistry functionalization, Adaptive wetting, Contact electrification, Ion transfer, Liquid-Solid Interface

*corresponding author: butt@mpip-mainz.mpg.de and wong@mpip-mainz.mpg.de

This file includes:

Supplementary Experimental Section

Supplementary Discussion

Supporting Figures S1-4

Supplementary Experimental Section

Atomic Force Microscopy

Scanning force microscopy (SFM) has been used to map the topography of surfaces. We performed the SFM studies with a soft tapping mode in air (Bruker, Dimension ICON). As the probe, and OTESPA silicon probe is used (Bruker, nominal spring constant 26 N/m and a nominal resonance frequency of 300 kHz).

Time-of-Flight Secondary Ion Mass Spectrometry (TOF-SIMS)

TOF-SIMS analysis was performed on pristine and drop-wetted surfaces using an IONTOF TOF.SIMS[5] NCS (Münster, Germany). The analyzer was operated at a cycle time of 150 µs corresponding to a mass range of 1-2070 u. 30 keV Bi_3^+ cluster ions were used as primary ions (current: 0.10 pA) rastering a field-of-view of 200 x 200 µm². A 2.5 keV Ar_{1000} gas cluster ion beam (current: 0.18 nA) was employed for depth profiling sputtering an area of 400 x 400 µm². The SurfaceLab software (Iontof, Münster, Germany) version 7.1 was used to evaluate the data. In TOF-SIMS, surfaces are bombarded with primary ions, resulting in the emission of secondary ions sputtered from the surface material. These ions are analyzed and the obtained mass spectra can be used to identify the composition of the material. The analysis of spectra from the pure chemical variants allows the identification of species which are indicative of the analyzed material (diagnostic ions). During depth profiling, one cycle of analysis is followed by a surface erosion step using a higher current ion gun. Repeating these alternating steps, a depth profile revealing the course of the ion signals with increasing depth is obtained. The diagnostic ions identified are fragments of the organic chains, which allow the identification of the presence of the materials in the layer system.

Supplementary Discussion

Surface Chemical Compositions: TOF-SIMS

To provide an improved understanding behind the chemical configuration of surfaces synthesized, depth profiling (See Supplementary Experimental Section) was performed using Time-of-Flight Secondary Ion Mass Spectrometry (TOF-SIMS). During profiling, the primary layers of PFOTS and TCPS produced fluorocarbon (CF_3^-) and hydrocarbon (C_xH_y) signatures respectively. The secondary layers of APTES and NTDET produced nitrogen-based fragments (C_3N^-). As the observed ions for TCPS were not unique due to the presence of C_xH_y fragments in PFOTS/APTES/NTDET, *i.e.* non-diagnostic, materials analysis was only performed with and alongside the base layer of PFOTS (Figure S2a-c, insets).

PFOTS-APTES and PFOTS-NTDET produce a mixed signature of diagnostic ions from both components. During the onset of depth profiling, the presence of both fluorocarbons (diagnostic ion CF_3^-) and amine-based hydrocarbons (diagnostic ion C_3N^-) was observed. We believe that despite the sequence of layers, fluorinated hydrocarbons may enrich at the surface, in order to minimize surface energy. This occurs in tandem with the preferential shielding of the surface energetic amine-groups (APTES/NTDET). The presence/absence of the different secondary layers (APTES/NTDET or without) did not affect the nature of decay for the fluorocarbon's diagnostic ion, CF_3^-. Therefore, this allows for a cross-comparison (Figure S2a-c, insets) between surfaces.

For PFOTS-APTES, the C_3N^- trace initially decayed at a lower speed compared to CF_3^-. Thereafter, both ions, CF_3^- and C_3N^-, decay at similar rates (Figure S2b, insets). For PFOTS-NTDET, a comparatively fast initial decay of C_3N^- occurs, in fact, similar to the decay of CF_3^-. Thereafter, the diagnostic ion C_3N^- decayed slower, at a similar gradient to APTES's C_3N^- (Figure S2c, insets). The decay profiles may indicate that amines in PFOTS-APTES are more evenly distributed throughout the entire depth as compared to PFOTS-NTDET.

In summary, TOF-SIMS analysis indicates that the hydrophobic profile of surfaces persists throughout the depth of the surfaces (purple data points, Figure S2 insets). In addition, the presence of proton acceptors (amines) is also prevalent in the sub-surface depths (blue data points, Figure S2 insets). As wetting is primarily influenced by the first 1-3 nm while electrification is deeper (3-5 nm),[1] this configuration is optimal for preserving hydrophobic wettability while integrating charging characteristics. Therefore, the primary layers of PFOTS and TCPS inhibit wetting and drop fragmentation while the secondary amine layers provide the desired slide electrification.

Supporting Figures

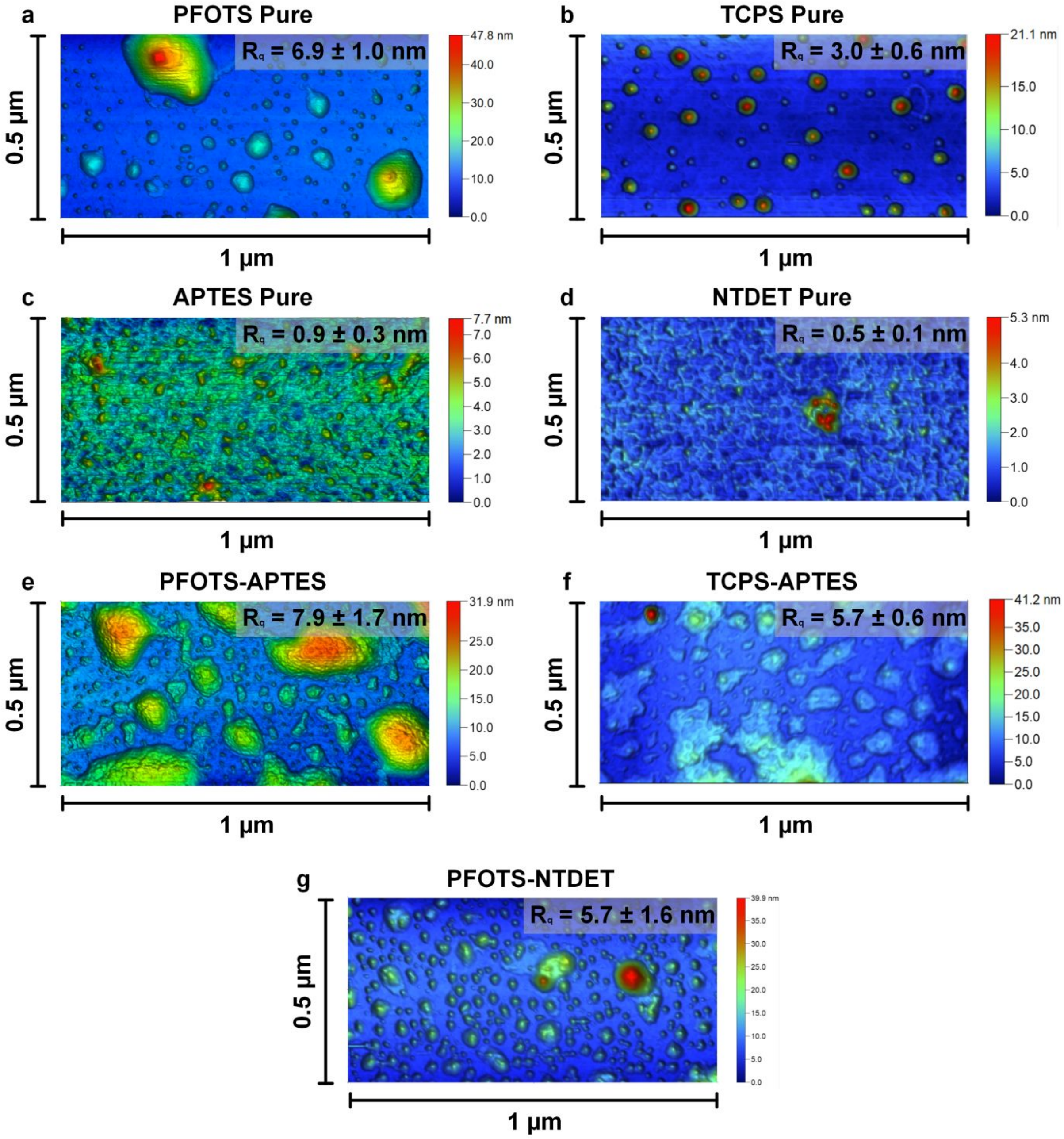

Figure S1. Atomic Force Microscopy Mapping (Magnified Images from Figure 1). Grids of 0.5 µm x 1.0 µm are presented to showcase high resolution imaging of the resulting surface roughness and nano-/micro-scopic features. The rms roughness, or $R_q$is presented (top right corner) alongside the topology maps. Test surfaces include the mono-variants: a) fluorocarbon, PFOTS, b) hydrocarbon, TCPS, c) mono-amine, APTES, d) multi-amine, NTDET, and the double-variants: e) PFOTS-APTES, f) TCPS-APTES, and g) PFOTS-NTDET.

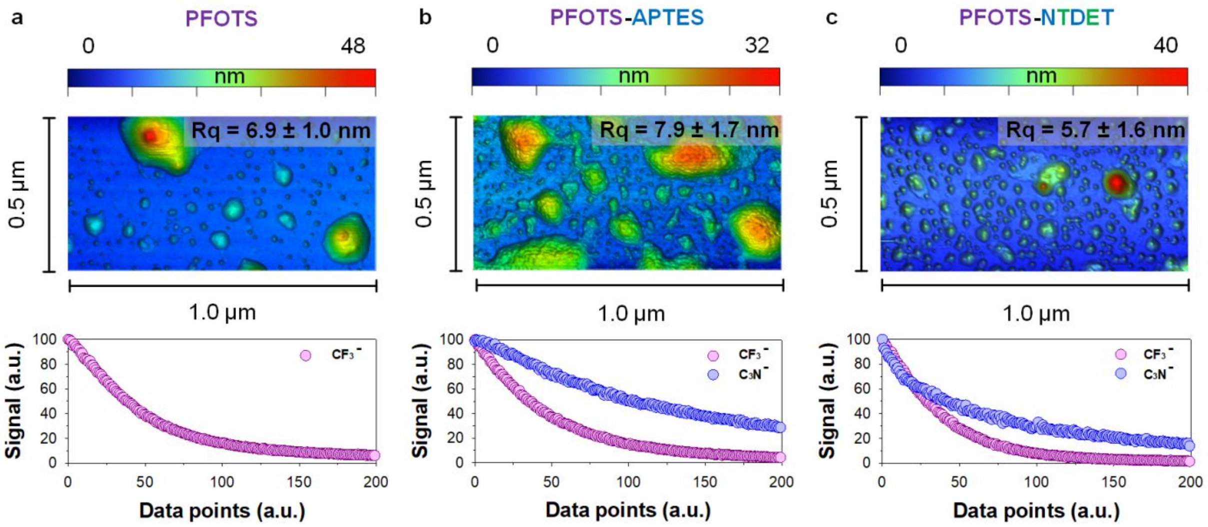

Figure S2. Morphology and surface chemistry characterization. Atomic force microscopy (AFM) and Time-of-Flight Secondary Ion Mass Spectrometry (TOF-SIMS) depth profiling was performed for a) PFOTS, b) PFOTS-APTES, and c) PFOTS-NTDET. Depth profiling is indicated as data points with progression of depth, but cannot be assigned physical values (*e.g.* Å) due to instrumentational limits. Signals obtained from the respective diagnostic ions are normalized. Topology mapping (AFM) is presented for pristine surfaces. Despite the presence of nanobumps and some variation in nanoroughness of surfaces synthesized by chemical vapor deposition (CVD), smoother- surfaces (R_q = 1-2 nm) do not experience differences in slide electrification properties from rougher- surfaces (R_q = 6-7 nm) (Figure S3).

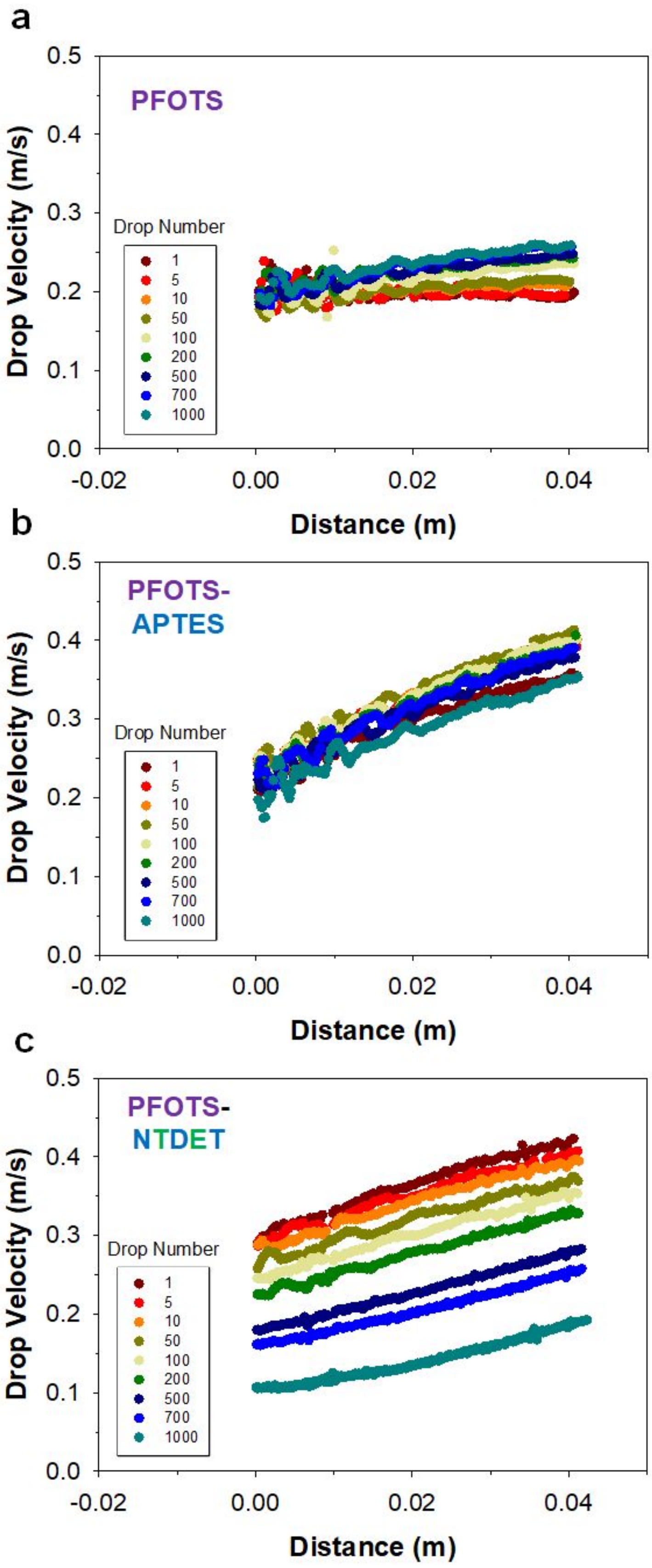

Figure S3. Drop mobility experiments (Repeats and Comparison). a) Positive drop mobility: PFOTS-only. b) Negative drop polarity: PFOTS-APTES. c) Adaptive drop polarity: PFOTS-NTDET.

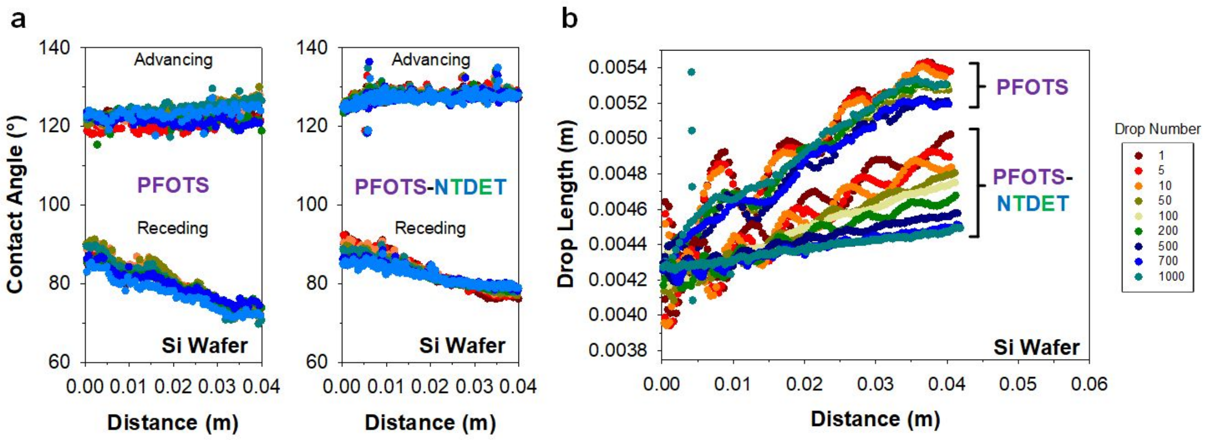

Figure S4. Influence of adaptation on drop profile (contact angle and drop length). a) The mobility changes described in the main manuscript occurs significantly despite fairly minor alterations to the measured advancing and receding contact angles. However, the method used (tilting plate) may be simply incapable of detecting the change of receding contact angles at a sub-10 µm scale. b) The drop length for PFOTS-NTDET during sliding changes considerably more than that for PFOTS. Together, changes in drop profile are likely caused by surface adaptation, attributed to surface chemistry and wetting alterations. These combined factors lead to changes in drop profile, hence adhesion, and mobility.

References

1. Henzler, M.; Göpel, W., *Oberflächenphysik des Festkörpers*. Vieweg+Teubner Verlag: 2013.
